# ING1 and 5-Azacytidine Act Synergistically to Block Breast Cancer Cell Growth

**DOI:** 10.1371/journal.pone.0043671

**Published:** 2012-08-20

**Authors:** Satbir Thakur, Xiaolan Feng, Zhong Qiao Shi, Amudha Ganapathy, Manoj Kumar Mishra, Peter Atadja, Don Morris, Karl Riabowol

**Affiliations:** 1 Department of Biochemistry and Molecular Biology, University of Calgary, Calgary, Alberta, Canada; 2 Southern Alberta Cancer Research Institute, Calgary, Alberta, Canada; 3 Department of Medicine and Oncology, University of Calgary, Tom Baker Cancer Center, Calgary, Alberta, Canada; 4 Department of Oncology and Clinical Neuroscience, University of Calgary, Calgary, Alberta, Canada; 5 Novartis Institute for Biomedical Research, Shanghai, China; University of Edinburgh, United Kingdom

## Abstract

**Background:**

Inhibitor of Growth (ING) proteins are epigenetic “readers” that recognize trimethylated lysine 4 of histone H3 (H3K4Me3) and target histone acetyl transferase (HAT) and histone deacetylase (HDAC) complexes to chromatin.

**Methods and Principal Findings:**

Here we asked whether dysregulating two epigenetic pathways with chemical inhibitors showed synergistic effects on breast cancer cell line killing. We also tested whether ING1 could synergize better with chemotherapeutics that target the same epigenetic mechanism such as the HDAC inhibitor LBH589 (Panobinostat) or a different epigenetic mechanism such as 5-azacytidine (5azaC), which inhibits DNA methyl transferases. Simultaneous treatment of breast cancer cell lines with LBH589 and 5azaC did not show significant synergy in killing cells. However, combination treatment of ING1 with either LBH589 or 5azaC did show synergy. The combination of ING1b with 5azaC, which targets two distinct epigenetic mechanisms, was more effective at lower doses and enhanced apoptosis as determined by Annexin V staining and cleavage of caspase 3 and poly-ADP-ribose polymerase (PARP). ING1b plus 5azaC also acted synergistically to increase γH2AX staining indicating significant levels of DNA damage were induced. Adenoviral delivery of ING1b with 5azaC also inhibited cancer cell growth in a murine xenograft model and led to tumor regression when viral concentration was optimized *in vivo*.

**Conclusions:**

These data show that targeting distinct epigenetic pathways can be more effective in blocking cancer cell line growth than targeting the same pathway with multiple agents, and that using viral delivery of epigenetic regulators can be more effective in synergizing with a chemical agent than using two chemotherapeutic agents. This study also indicates that the ING1 epigenetic regulator may have additional activities in the cell when expressed at high levels.

## Introduction

Breast cancer accounts for approximately 45,000 cancer related deaths in North America per year. They are biologically heterogeneous, have distinct natural histories and respond variably to established therapies. Therapies available for treatment of breast cancer include surgical removal, radiation therapy, chemotherapy, immunomodulatory therapy, hormone therapy in cancers overexpressing hormone receptors like estrogen receptors (ER) and progesterone receptors (PR) and targeted biological therapy such as Trastuzumab in HER2/neu-positive patients. Recently, epigenetic mechanisms have become attractive targets for cancer therapy since drugs have been developed that have a wide therapeutic window, partially due to the fact that epigenetic changes are reversible, meaning that off target effects should be minimal and reversible upon cessation of treatment. As a consequence, several drugs that affect epigenetic pathways have been approved for cancer treatment and more are currently in clinical trials [1].

ING1 (INhibitor of Growth 1) is a type II tumor suppressor that was identified using PCR mediated subtractive hybridization between normal and cancerous breast epithelial cells followed by a functional screen for tumor induction [2]. ING proteins are down regulated in both familial and sporadic breast cancers [3], [4], [5] and overexpression of ING1 can induce cell cycle arrest and apoptosis [6]. INGs also function in histone acetylation [7], [8] acting as stoichiometric members of HAT and HDAC complexes [9]. Reading the histone code and the subsequent contribution of ING proteins to modulating local chromatin structure by altering histone H3 and H4 acetylation likely accounts, in large part, for ING’s regulation of gene expression. Based upon frequent down regulation of INGs in cancer cells and their biological relationship with p53, loss of INGs may also affect breast cancer cell response to chemotherapeutic agents as suggested for vincristine in brain tumors [10] and for paclitaxel and etoposide in osteosarcoma cells [11].

Cancer cells accumulate genetic and epigenetic changes that alter gene expression to drive tumorigenesis [12] and epigenetic silencing of tumor suppressor, cell cycle, differentiation and DNA repair genes contributes to tumorigenesis [13]. Epigenetic abnormalities that are commonly found in human tumors can often be reversed by pharmacologic inhibitors, such as HDAC inhibitors and DNA methylation inhibitors. One of the histone modifications showing promise as a target for cancer treatment is acetylation [14]. Acetylation is increased by HDAC inhibitors like sodium butyrate, resulting in decondensation of heterochromatic DNA and increased sensitivity to DNAse [15]. HDAC inhibitors that are showing promising effects in clinical trials such as LBH589 (Panobinostat), are pan-deacetylayse inhibitor, being capable of inhibiting all HDACs that require Zn as a cofactor [16]. It is interesting to note that the molecular target of the HDAC inhibitor suberoylanilide hydroxamic acid (SAHA) was recently identified as ING2, a stoichiometric member of the Sin3 HDAC complex [17], suggesting that targeting of the INGs themselves may prove useful in combination with other chemotherapeutic agents.

DNA methylation can be modified pharmacologically and cancer was the first disease proposed as a therapeutic target [18]. DNA methylation is altered in cancers by hypermethylation of tumor suppressor genes, aberrant expression of DNA methyl transferases (DNMTs) and hypomethylation of unique genes and repetitive sequences. The three most commonly used catalytic inhibitors of DNMTs and hence, DNA methylation, are the nucleoside analogs 5-azacytidine (5azaC), 5-aza-deoxycytidine (5azaCdR) and Zebularine. 5azaC, and its deoxy analog 5azaCdR are FDA approved for treatment of myelodysplastic syndromes [19]. When a 5′-azacytosine ring replaces cytosine, DNMT is trapped on DNA [20] resulting in passive loss of DNA methylation in the nascent strand. While positive responses with tolerable adverse effects were reported in clinical trials for hematological malignancies [21], success in solid tumors has been elusive, which may be due to ineffective delivery, dosing or scheduling [22]. Different strategies for combining 5azaC with other chemotherapeutic agents or chromatin modifiers such as HDAC inhibitors are currently being tested in solid tumors.

Several of the ING proteins can inhibit the growth of cancer cells *in vitro* and *in vivo* when overexpressed using adenoviral vectors [23], [24], [25], [26], [27], [28]. The ING proteins might be particularly useful to enhance chemosensitivity in combination with agents like etoposide and doxorubicin [29]. These reports suggest that ING1 may act to restore tumor suppressor functions and augment the effects of various chemotherapeutic agents when used in combination. The aim of this study was to determine the potential of combining ING1 gene therapy with different classes of epigenetic drugs such as HDAC inhibitors and DNA methyl transferase inhibitors, with the hypothesis that some combinations might demonstrate synergy in eliminating cancer cells *in vitro* and *in vivo* using a murine xenograft model.

## Materials and Methods

### Animals and Ethics Statement

Thirty 6–8 week old female CB-17 Fox Chase SCID mice were purchased from Charles River Laboratory (Montreal, Quebec, Canada) and maintained under a level II biohazard containment in the local animal facility. All procedures and treatment were reviewed and approved by the University of Calgary Animal Care Committee and conformed to guidelines of the Canadian Council on Animal Care (CCAC; http://www.ccac.ca/en_/standards/guidelines/additional/vol2_mice).

### Cell Culture

Established human breast cancer cell lines MCF7 (HTB22), BT20 (HTB19), MDA-MB435S (HTB129), SKBR3 (HTB30), T47D (HTB133), ZR-75-1 (CRL1500), BT474 (HTB20), Hs578T (HTB126), and MDA-MB468 (HTB132) and the immortal human mammary epithelial cell line MCF10A (CRL10317) were purchased from the ATCC. It is worth noting that MDA-MB-435S cells may be derived from a melanoma. Normal human breast epithelial cells Hs578Bst (HTB125) [30] were a gift from Martha Stampfer. MCF10A and all breast cancer cells were cultured in high glucose Dulbecco’s Modified Essential Media (H-DMEM) supplemented with 10% FBS, 0.1 mg/ml streptomycin and 100 U/ml penicillin. Hs578Bst cells were cultured in L-DMEM supplemented with 30 ng/ml and antibiotics. All cells were maintained in a humidified atmosphere at 37°C and 5% CO_2_ and tested negative for mycoplasma. Culture media were changed every 2–3 days.

### Generation of Adenoviral Constructs

Adenoviral constructs were generated using a modified “pAdEasy” system [31]. ING1b and ING2 were subcloned into pAdTrack-CMV, which contains a separate EGFP expression cassette and were recombined with pAdEasy-1 in BJ5183 *E. coli* cells. Recombinant clones were screened and subsequently verified by a series of enzymatic digestions and sequencing. Recombinants were re-amplified in XL-Blue (Clontech), linearized by PacI (NEB) and transfected into HEK 293 cells for packaging. ING1 adenovirus contained ING1 and GFP under the control of separate promoters. Viral clones were plaque purified, selected for expression, amplified and purified by CsCl_2_ gradient centrifugation. Viral titers were routinely estimated to ensure accurate viral concentrations. Adenoviral infections were optimized by titrating virus to identify multiplicities of infection (MOIs) of cells giving >95% infectivity as monitored by GFP expression and which showed minimal effects from control virus (Ad-GFP) infection. No toxicity was observed when adenoviruses were used at these MOIs.

### Treatment Protocol for Epigenetic Drugs

MDA-MB468 cells were cultured for 24 hours and then treated with LBH589 (Novartis Pharmaceuticals) or 5azaC (Sigma) at determined IC_50_ of 100 nm for 15 hours and 40 µM for 48 hours, respectively. Cells were infected with Ad-ING1b (30 MOI) after pretreatment with the epigenetic drugs, and were harvested 24 hours post infection. For 5azaC treatment, media containing fresh 5azaC were changed every 24 hours. LBH589 and 5azaC were prepared as 5 mM and 100 mM stocks in DMSO and PBS respectively, and stored at −80°C until use.

### MTT Assays

Approximately 5×10^4^ MDA-MB468 cells were plated per well in a 96 well plate and treated with various concentrations of LBH589, 5azaC and various titres of Ad-GFP or Ad-ING1b, alone or in combinations. At the end of treatments, 50 µl of MTT (3-(4,5-dimethylthiazol-2-yl)-2,5-diphenyltetrazolium bromide) was added to each well from a 5 mg/ml stock in PBS. The plates were then incubated for 4 hours at 37°C in the dark. The supernatant was aspirated and formazan crystals were solubilized in 100 µl DMSO at 37°C for 10 min with gentle agitation. Absorbance from the plates was read at 570 nm with a Bio-Rad microplate reader. Percent growth inhibition was calculated by the formula (OD_control_ - OD_treated_)/OD_control_ ×100.

### Apoptosis and Cell Viability Assays

Exponentially growing cells were seeded into fresh 10 cm plates at ∼10% confluence 16–18 hours prior to infection. 48 hours after infection cells were harvested, washed in PBS +5 mM EDTA and fixed in 70% ethanol at 4°C for 30 min. Cells were washed twice with PBS +5 mM EDTA followed by staining in propidium iodide (PI) solution (50 µg/ml PI, 20 µg/ml RNase) (Sigma) in PBS in the dark at RT for 20–30 min. Samples were subsequently analyzed by flow cytometry (Flow Cytometry Facility at University of Calgary) within one hour. An Annexin V-PE/7AAD kit (BD Pharmingen) was used following the manufacturer’s instructions to identify apoptotic cells by a FACScan flow cytometer in combination with BD FACSDiva Software (Becton-Dickinson). Viability of cells was assessed by trypan blue exclusion assay. The floating dead cells in the medium and cells that remained attached to the plates were collected by trypsinization and counted using a hemocytometer in the presence of 0.4% trypan blue reagent (Sigma). All experiments were done in triplicate.

### Combination Index Calculations

The modes of interaction of 5azaC with LBH589 and ING1b with 5azaC or LBH589 were analyzed using CalcuSyn software (Biosoft, Cambridge, United Kingdom). The software is based on the calculations developed by Chou and Talalay [32], which allows the evaluation of interactions between 2 or more drugs. The combinations of Ad-ING1b (15, 25, 35, 45 and 55 MOI) with 5azaC (20, 30, 40, 50 and 60 µM) or LBH589 (50, 75, 100, 125 and 150 nM) were tested at different ratios and inhibition of cell growth was determined by MTT assay. The software utilizes a multiple drug-effect equation derived from an enzyme kinetics model in which the output is represented as combination indices (CI) and/or isobologram analysis. Calcusyn defines synergy between two drugs when the CI value is <1. The extent of synergism/antagonism may also be determined based on the CI value. In brief, CI values between 0.9 and 0.85 suggest a moderate synergy, whereas those in the range of 0.7 to 0.3 are indicative of clear synergistic interactions between the drugs. On the other hand, CI values in the range of 0.9 to 1.10 would suggest an additive effect while those >1.1 suggest antagonism.

### Western Blotting

MDA-MB468 cells were treated with 5azaC, followed by infection with the indicated adenoviral constructs and harvested 24 hours after infection. Cells were washed with PBS, lysed in SDS loading buffer, sonicated on ice and following PAGE, samples were transferred to nitrocellulose membrane (Millipore) by electrophoresis and blotted with α-ING1 monoclonal (SACRI Antibody Services), α-PARP (Santa Cruz), α-γH2AX (Millipore), α-Caspase-3 and α-β actin (Cell Signaling) antibodies.

### Tumor Implantation and Treatment

To establish subcutaneous tumors, actively growing MDA-MB468 breast cancer cells were harvested and 7×10^6^ cells in 100 µl PBS were injected into the mammary fat pads of mice. The lesions were allowed to grow until their average sizes were approximately 5 mm ×5 mm (about 2 weeks). The mice were then randomized into 6 groups for various treatments including PBS vehicle control, Ad-GFP control, Ad-ING1b, 5azaC, Ad-GFP plus 5azaC, and Ad-ING1b plus 5azaC combination groups. Treatment started at day 1, 2×10^8^ PFU of Ad-GFP or Ad-ING1 were given intratumorally (it) twice a week for a total of 10 treatments. 5azaC was administered intraperitoneally (ip) every other day (3 times a week) at 5 mg/kg for a total of 15 injections (5 weeks). Tumors were then re-challenged (2×10^9^ pfu) for another 3 weeks in the same pattern from day 57 to 77 when the tumors in the combination group showed signs of rapid growth. Tumor size and body weight were recorded twice weekly. Tumor cross-sectional area was calculated by multiplying the length × width and tumor volume was calculated by cubing the mean value of length and width. Dose of 5azaC to be used was determined in preliminary trials testing different doses of 5azaC versus tumor size and total animal body weight. As shown in Figure S6, 5 mg/kg was optimal for having no effect on body weight but an effect on inhibiting tumor growth.

### Statistical Analyses

Data are expressed as mean ± SEM. Statistical significance was assessed by one-way ANOVA and Tukey’s Multiple Comparison test. For analyzing results from experiments involving combination of different epigenetic drugs, two-way ANOVA with Bonferroni’s Multiple Comparison test was used. Bars represent the average of three independent trials showing standard error of mean for all figures. A value of P<0.05 was considered significant and represents significance compared with untreated controls, unless indicated otherwise.

## Results

### ING1b and ING2 Act Independent of p53 Status

**Figure 1:**
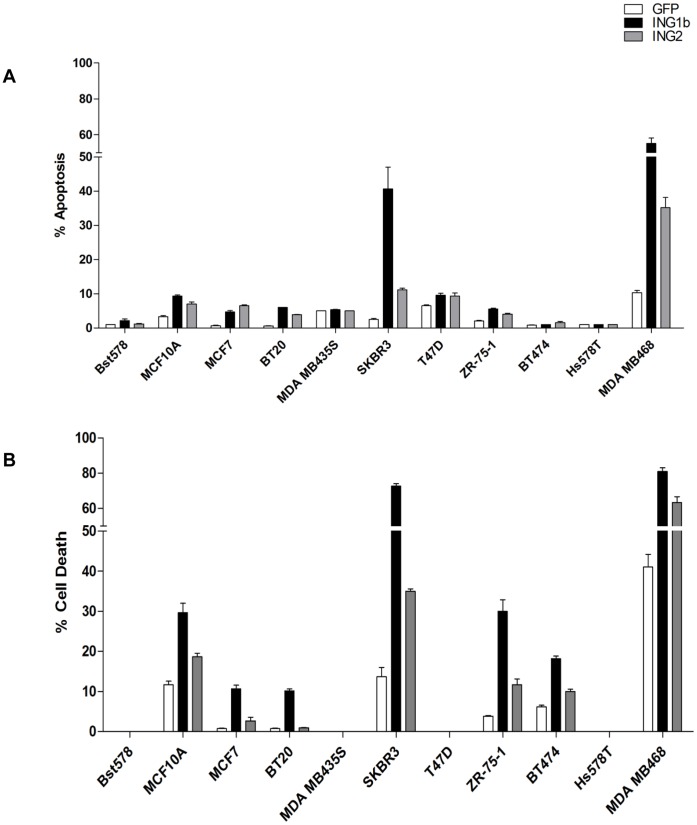
Cell Death and Apoptosis in response to ING1 and ING2. The indicated breast cancer cell lines were grown and tested for the ability of adenoviral constructs encoding GFP, GFP plus ING1b or GFP plus ING2 expressed from separate promoters to **A**) induce apoptosis as estimated by propidium iodide staining for sub-G1 DNA content, or **B**) induce cell death as estimated by cell survival (Coulter counting). Results were obtained using an MOI of 10, 48 hours after infection. The MDA-MB468 and SKBR3 lines were most susceptible to ING at this MOI and other lines showed increased susceptibility at higher MOIs (data not shown). Normal Bst578 cells were fully resistant to this concentration of virus. Uninfected cells were used as controls to normalize other cell numbers against.

ING1 expression is reduced in breast tumors and breast cancer cell lines [2], [3], [4], [5], [33] but few studies have tested the effects of increasing ING1b in breast cancer cells. In contrast, induction of apoptosis by other INGs has been reported in many cancer types [34], [35], [36], [37], [38], [39] and in normal diploid fibroblasts [8], [40] and some reports suggest that ING1 requires p53 activity to induce apoptosis [35], [41]. To further test this idea, nine breast cancer cell lines and two non-tumorigenic breast epithelial cell strains with different growth rates (Figure S2) and with varying ER, p53 and HER2/neu status (Table 1) were infected with GFP, GFP-ING1b or GFP-ING2 to see if ING proteins affected breast cancer cells in a p53-sensitive or growth rate-dependent manner. MDA-MB468 and SKBR3 cells were most susceptible to both ING1b and ING2 compared to other cell lines (Figure 1) suggesting that neither growth rate nor p53 status strongly affected the ability of ING1 to induce apoptosis.

**Table 1:** Characteristics of breast epithelial cell lines examined.

Cell Lines	Cell Source	Tumorigenic (Nude Mice)	ER Status	p53 Status	HER2/neu overexpression
Bst578	Normal Tissue	No	+	Wild Type	No
MCF10A	Fibrocystic disease (immortalized)	No	+	Wild Type	No
MCF7	Adenocarcinoma (pleural effusion)	Yes	+	Wild Type	No
BT20	Adenocarcinoma (pleural effusion)	Yes	−	Mutant	Yes
MDAMB435	Ductal carcinoma (pericardial effusion)	No	−	Mutant	No
SKBR3	Adenocarcinoma (pleural effusion)	Yes	−	Mutant	Yes
T47D	Ductal carcinoma (pleural effusion)	Yes	+	Mutant	No
ZR-75-1	Ductal carcinoma (ascites)	Yes	+	Wild Type	No
BT474	Ductal carcinoma	Yes	+	Mutant	Yes
Hs578T	Ductal carcinoma	No	−	Mutant	No
MDAMB468	Adenocarcinoma (pleural effusion)	Yes	−	Mutant	No

Bst578 is a normal primary epithelial cell strain and MCF10A is an established, but phenotypically normal cell line. MCF7 is also known to harbor a caspase 3 mutation that makes it relatively resistant to apoptosis. MDA-MB-435S cells may be a melanoma cell line.

A previous study reported a positive association between ING1b and ER levels in breast cancer tissues [3], and ING1b stimulates the transcriptional activity of ERα [42], [43]. Thus, we asked if ER negative breast cancer cell lines that are at a more advanced stage would be more sensitive to exogenous ING1 than ER positive cells. Our results support this idea since both SKBR3 and MDA-MB468 are ER negative and they exhibited the greatest sensitivity to both ING1b and ING2. ING1 most effectively induced apoptosis and cell death in cells with mutant, rather than wild type p53 (SKBR3 and MDA-MB468), but was also able to induce cell death in MCF10A and ZR-75-1 that encode wild type p53. Thus, no clear association between ING1 killing efficiency and p53 status was seen, nor was any association between killing efficacy and HER2/neu overexpression noted.

### ING1b Enhances the Efficiency of Cell Killing by Epigenetic Drugs

**Figure 2:**
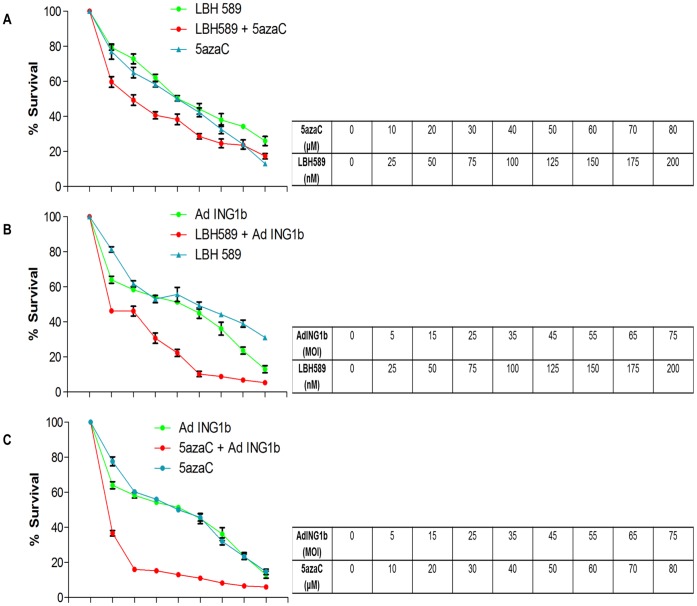
Cell death in MDA-MB468 cells in response to ING1b and epigenetic chemotherapeutics. MDA-MB468 cells were grown and treated with various concentrations of **A**) LBH589 and 5azaC alone or in combination, or **B,C**) in combination with adenoviral constructs expressing GFP plus ING1b at various MOIs. In combination treatments, cells were pretreated with LBH589 for 15 hours or 5azaC for 48 hours and then infected with adenoviral constructs and analyzed after 24 hours. The levels of cell death induced by these treatments were estimated by MTT assay. The combination of 5azaC with ING1b shown in panel C was more effective in inducing cell death in MDA-MB468 cells at all concentrations compared to other agents tested singly or in combination. Two way ANOVA with Bonferroni’s multiple comparison test was used for calculating P values (P<0.001 for both combination treatments shown in panels B&C compared to single agents).

Cancer cells frequently show altered DNA methylation and histone modifications such as histone acetylation, resulting in deregulation of gene transcription [44]. To test whether targeting these two epigenetic pathways simultaneously would induce synergistic cell killing, MDA-MB468 cells that are sensitive to ING1 were treated with LBH589 and 5azaC independently and in combination. As shown in Figure 2A, a weak additive effect was noted. Since ING1 enhanced paclitaxel- and etoposide-induced apoptosis in osteosarcoma cells [45], we asked if ING1b could also enhance toxicity of 5azaC and the third generation HDAC inhibitor LBH589. ING1b enhanced the ability of both LBH589 and 5azaC to induce cell death better than when combining LBH589 with 5azaC and ING1b was more effective in combination with 5azaC. This was not a result of using a relatively more effective dose of 5azaC, since 5azaC and LBH589 were used at concentrations that had similar effects upon survival individually. To determine if this relationship held in another cell line, T47D cells, which are very resistant to ING1-induced cell death (Figure 1B), were tested using the same protocol. As seen in Figure S3, although viral titres used were significantly higher and were tested through an even larger range, the combination of 5AzaC and ING1b was again, most effective in killing cells. These results indicate that targeting two different epigenetic mechanisms using a biological agent in combination with a chemical agent is more effective that using two chemical agents in inducing cell death in breast cancer cells, but the absolute effects are greatest in cells more sensitive to ING1.

### ING1b Acts in Synergy with 5azaC to Induce Cell Death

**Figure 3:**
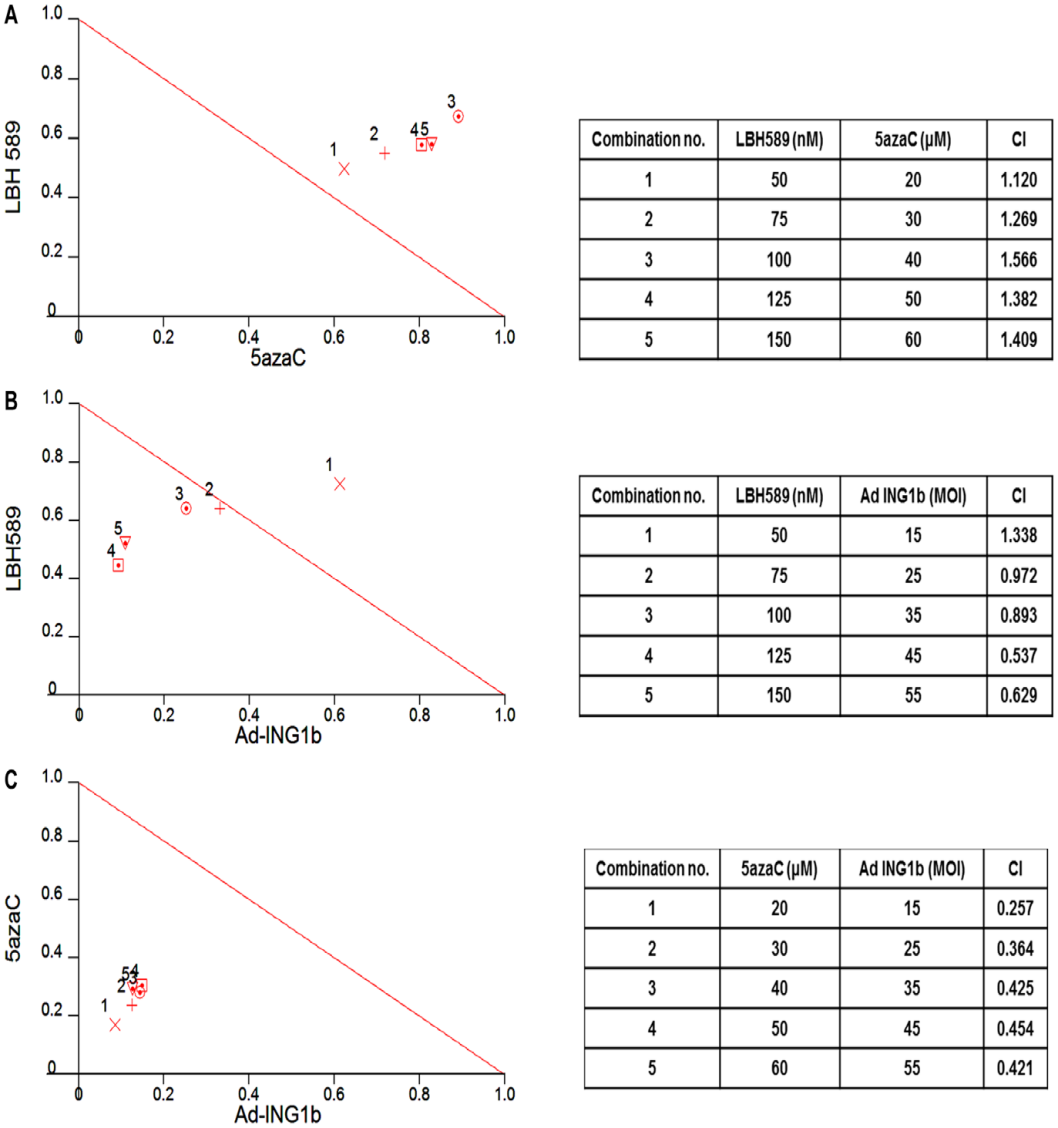
Combination Indices of ING1b with epigenetic chemotherapeutics. MDA-MB468 cells were treated with combinations of **A**) LBH589 plus 5azaC, **B**) adenoviral vector expressing GFP plus ING1b and LBH589 or **C**) adenoviral vector expressing GFP plus ING1b plus 5azaC at various concentrations and Combination Indexes were determined using CalcuSyn software. The Isobologram analysis showed that 5azaC plus Ad-ING1b showed the highest degree of synergy in inducing cell death of the combinations tested. Data used to generate this graph represent a subset of the combinations shown in Figure 2.

To quantitatively determine how much more effective targeting two separate, versus a single epigenetic pathway was, we assessed interactions using Normalized Isobolograms and Combination Index (CI) values generated using CalcuSyn software. Cells were pretreated with various doses of 5azaC or LBH589 and then treated with the other chemotherapeutic or infected with ING1b adenovirus. Intensities of interactions were determined based upon the CI value generated by the software with particular combinations of drugs and ING1b. Treating with both epigenetic drug agents gave a less than additive effect (Figure 3A). In contrast, the combination of ING1b with LBH589 ranged from non-synergistic to synergistic (CI 0.9-0.5) when both ING1b and LBH589 were used at higher doses (Figure 3B). The combination of ING1b with 5azaC showed clear synergy (CI 0.4-0.2) at all concentrations tested (Figure 3C). Plotting of isobolograms with data generated from ING1-resistant T47D cells (Figure S4A–C) confirmed that the most synergy in T47D cell killing was seen between ING1b and 5AzaC (Figure S4C) as previously noted for cells of the MDA-MB468 line, indicating that this effect was not dependent upon the absolute sensitivity of cells to ING1-induced death.

**Figure 4:**
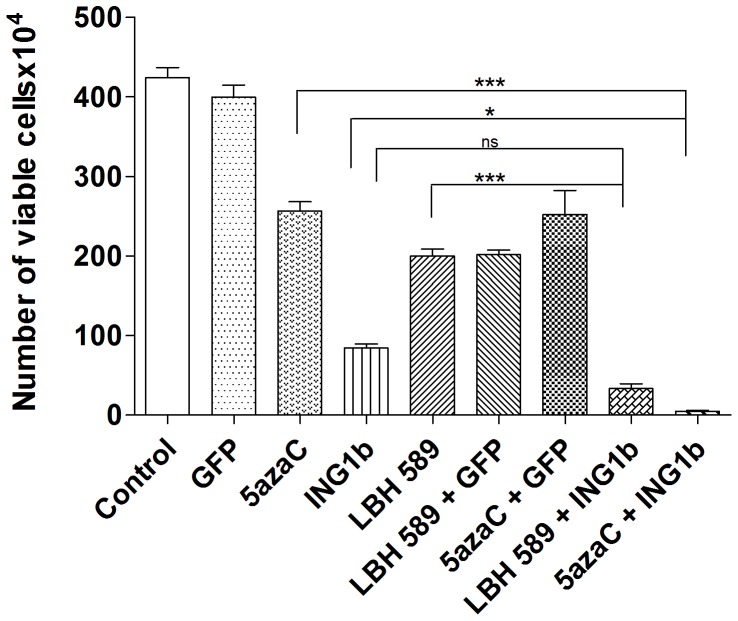
Effects of ING1b in combination with epigenetic chemotherapeutics. MDA-MB468 cells were treated alone or with the combinations of virus (30 MOI) and 5azaC (48 hours, 40 µM) or LBH589 (15 hours, 100 nM) indicated. Untreated cells and cells infected with Ad-GFP (30 MOI) alone served as controls. Cells were harvested 48 hours after infection and stained with Trypan Blue. Cell number was determined by counting the number of unstained cells using a haemocytometer. One-way ANOVA and Tukey’s multiple comparison post-tests were performed to calculate P values (*** indicates P<0.0001 compared to the control).

To further test this using an independent assay, cells were pretreated with 5azaC or LBH589 at IC_50_ concentrations and subsequently infected with adenovirus (30 MOI) expressing GFP or GFP plus ING1b. The treatment groups with combinations of ING1b with 5azaC and ING1b with LBH589 showed significant decreases in the number of viable cells (P<0.0001) 48 hr after infection as estimated by trypan blue staining (Figure 4). Again, the combination of ING1b plus 5azaC was most efficacious and synergistic, virtually eliminating viable cells. This is consistent with the idea that targeting two pathways eliminates cancer cells more effectively than targeting one pathway with two agents, and demonstrates that adenoviral infection does not induce cell death at these MOIs alone, or when combined with 5azaC or LBH589.

### ING1b Plus 5azaC Induce Apoptosis and DNA Damage

**Figure 5:**
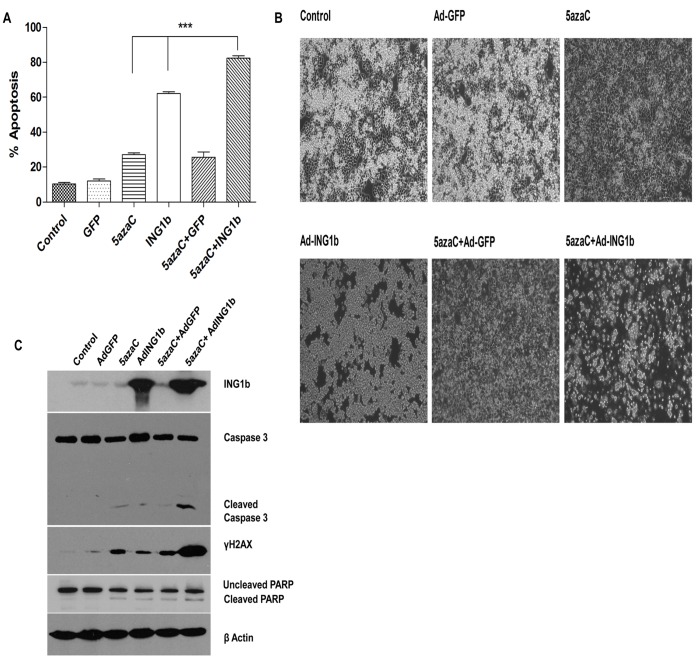
Apoptosis in response to 5azaC and ING1b in MDA-MB468 cells. **A**) Cells were pretreated with 40 µM 5azaC for 48 hours and then infected with adenoviral constructs expressing GFP or ING1b plus GFP (30 MOI). Untreated cells and cells infected with only Ad-GFP or Ad-ING1b plus GFP served as controls. Cells were harvested 24 hours later, stained for Annexin V and the percentage of the cell population undergoing apoptosis was estimated by determining the percentage of Annexin positive cells by flow cytometry. Cells treated with a combination of 5azaC and Ad-ING1b plus GFP showed higher amount of apoptosis induced in comparison to controls. One way ANOVA and Tukey’s multiple comparison post-test were performed to calculate P values comparing treated to untreated control cells (*** indicates P<0.0001). **B**) Clear morphological changes were noted in cells 24 hours after treatment with Ad-ING1b alone, or in combination with 5azaC. The combination blocked cell growth and induced morphological changes consistent with apoptosis in the great majority (99%+) of cells examined. Infection with Ad-GFP had little effect upon cell number or shape while 5azaC inhibited cell growth but was not effective in inducing an apoptotic phenotype in the majority of cells. **C**) Cells treated with the indicated agents for 48 hours were harvested in Laemmli sample buffer, lysates were boiled and equal amounts of protein from each sample were electrophoresed and blotted with the indicated antibodies.

Cells treated with combinations of 5azaC, control and ING1 adenovirus were analyzed for Annexin V binding as a marker of early apoptosis (Figure S5). 5azaC and ING1b caused significantly higher percentages of apoptotic cells in comparison to the 5azaC only, ING1b only and the GFP controls (Figure 5A; P<0.0001), and this effect was reflected by reduced numbers of cells showing cell morphology consistent with apoptosis (Figure 5B). MDA-MB468 cells appeared to be very sensitive to ING1b-induced early apoptosis events as estimated by Annexin V binding, which may explain an apparently additive, rather than synergistic effect when combined with 5azaC in this experiment. If analysis of the entire population of cells is done in the flow cytometry analysis, rather than omitting cells that have been killed by the treatment, the combination of 5azaC and ING1b again shows clear synergy (Figure S7). Most cells treated with Ad-ING1b showed morphology reminiscent of apoptosis, but cells survived longer than those treated with 5azaC plus ING1b. Fewer cells were present on plates treated with 5azaC due to inhibition of cell cycle progression [46].

Apoptosis involves initiation, effector and execution phases [47]. To better determine the progression of the apoptotic process in response to these agents, we evaluated the status of caspase-3 and PARP, which act at later stages of apoptosis. A significant increase in the amount of cleaved caspase-3 was noted in cells treated with the combination of ING1b and 5azaC compared to treatment with single agents (Figure 5C; Figure S8). A similar trend was seen in the ratio of cleaved:uncleaved PARP. 5azaC is also known to induce DNA double strand breaks in cells [48], which can be estimated by evaluating levels of phosphorylated histone variant γH2AX. A synergistic increase in the level of γH2AX was observed in response to 5azaC plus ING1b compared to 5azaC or ING1b alone. This may be due to the fact that 5azaC has been shown to act synergistically with Bortizamide, a proteasome inhibitor and with Doxorubicin in inducing DNA damage [48]. ING1b was reported to be able to affect proteasomal degradation of several proteins including p53 [49] and Doxorubicin specifically affects ubiquitination of a subset of proteins [50]. Thus, it is tempting to speculate that convergent effects of 5azaC and ING1b in blocking proteasomal degradation may result in increased DNA damage, through a currently undefined mechanism, that may or may not involve the reactivation of major tumour suppressor genes such as p53 [36], or Rb [51]. These data provide the first evidence that ING1b exacerbates 5azaC-induced DNA damage.

### Ad-ING1b Plus 5azaC Significantly Reduce Tumor Size in a Mouse Xenograft Model

**Figure 6:**
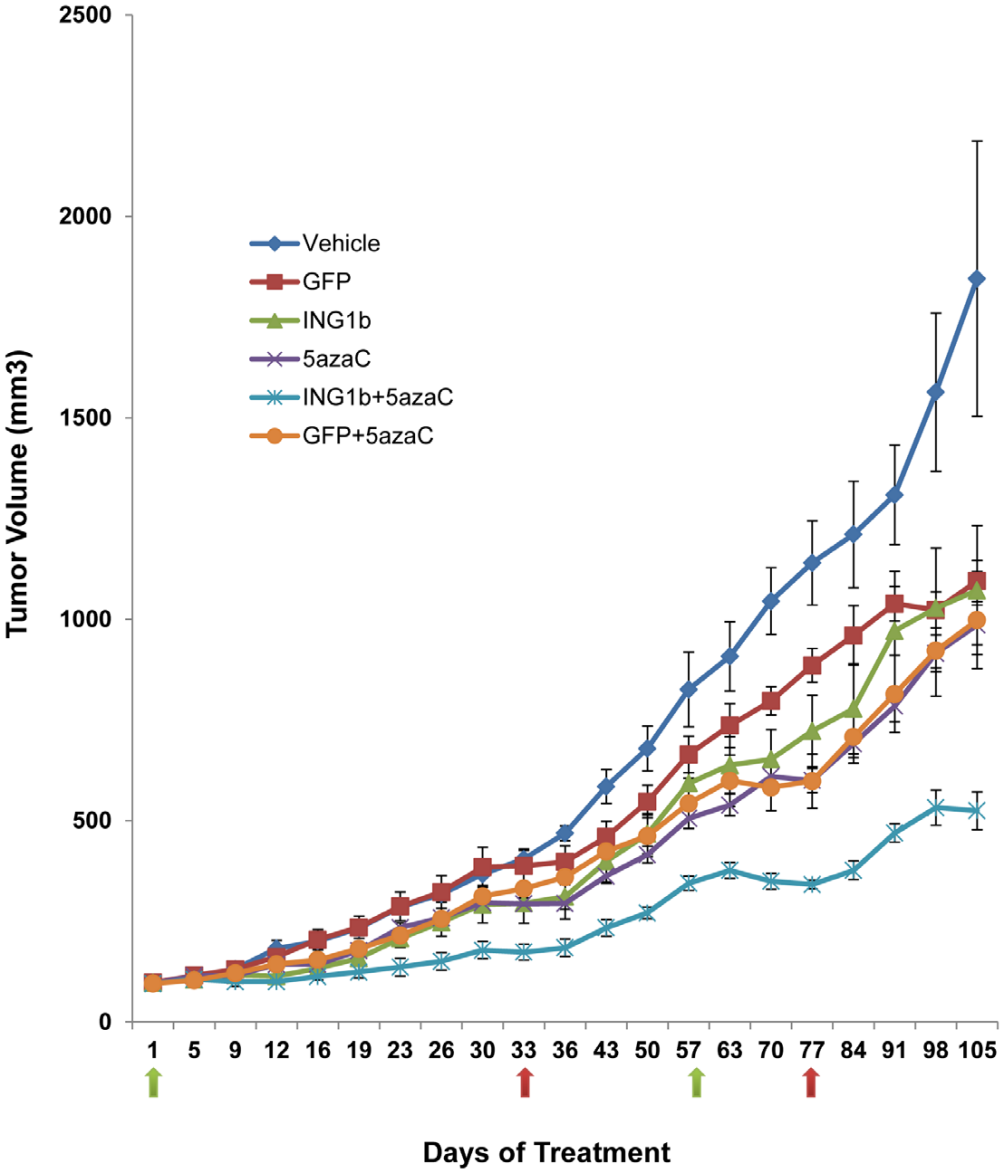
AdING1b plus 5azaC significantly reduce tumor volume *in vivo* in a mouse xenograft model. Approximately 7×10^6^ MDA-MB468 cells/animal were injected into the mammary fat pads of mice and in two weeks when tumors reached a size of 5 mm ×5 mm ×5 mm, treatment was begun (green arrow at day one). Viral constructs (2×10^8^ PFU, calculated to provide an MOI of 1–5) were injected intratumorally twice a week for 5 weeks (10 injections) and 5azaC was injected intraperitoneally three times a week for 5 weeks (15 injections). Treatments were halted at day 33 (first red arrow on the abscissa) and resumed at day 57 with 2×10^9^ PFU of virus and the same concentration of 5azaC. Given the larger tumor volume, this was calculated to increase the MOI by ∼3-fold over the initial MOI. As noted by the decrease in tumor volume over days 57–77, tumors had not developed resistance to the combination treatment and regressed in response to higher viral titers. No adverse reactions were noted to this concentration of virus, which suggests that the relatively conservative amounts of virus could be increased to eliminate tumors. Each time point represents the average value of five animals per group.

We next tested for ING1b-5azaC synergy using an *in vivo* xenograft tumor model. Pilot experiments determined that treatment with 5 mg/kg of 5azaC affected tumor growth but not animal body weight (Figure S6). MDA-MB468 cells were injected into SCID mice to generate tumors and when tumors reached 125 mm^3^, treatments with combinations of 5azaC and ING1b were started. Animals were treated from day 1 through 33, monitored in the absence of treatment from day 33 through 57, and were then treated from day 57 through 77 with 10-fold more virus (a 3-fold increase in MOI due to larger starting volume of tumor) to see if tumors acquired resistance as often seen in response to other agents [52]. As shown in Figure 6, the combination of 5azaC and ING1b was the most effective in inhibiting tumor growth at the lower MOI of Ad-ING1b and tumors decreased in size in response to injection at the higher MOI used. Injected animals showed no adverse side effects at the higher level of virus. The growth of the tumors was monitored until day 105 at which point animals were sacrificed according to animal care guidelines. These data indicates that synergy between ING1b and 5azaC was maintained in vivo, that cells did not acquire resistance to virally expressed ING1b, and that higher viral titres were effective in reducing tumor volume. The latter observation underscores the importance of optimizing viral dosage *in vivo* versus *in vitro*, where much more effective killing was observed at relatively lower MOI.

## Discussion

In this study we have found that ING1b and ING2 proteins differentially induce cell death and apoptosis in breast cancer cell lines compared to normal breast epithelial cells. This activity does not depend upon the status of p53 or HER2/neu levels, but increased efficiency was seen in cells that were ER negative. In contrast to the lack of synergy and even antagonism seen when using the chemical agents 5azaC and LBH589 to target two distinct epigenetic pathways in cancer cells, the efficiency of ING1b mediated cell death increased significantly when it was used in combination with both LBH589 and 5azaC. However, synergy was significantly greater with 5azaC, which targets a distinct epigenetic mechanism compared to ING1. Treatment with ING1b plus 5azaC induced apoptosis by several criteria, and also acted to synergistically induce DNA damage as estimated by increased levels of γH2AX. This combination also inhibited tumor growth in subcutaneously xenografted tumors in mice. The MOI that was optimal for elimination of cells *in vitro* when used with 5azaC (MOI = 30; Figure 4) was insufficient to completely block tumor growth *in vivo*, although an MOI of 90 was able to cause tumor regression with no obvious side effects. Based upon this logic of targeting different pathways, we speculate that, when used with viral constructs encoding proteins that affect DNA methylation, LBH589 would synergize better than 5azaC. Such combinations may act more effectively since both increasing histone acetylation and demethylating promoters may be needed to reactivate the expression of genes inactivated in cancer cells such as tumor suppressors involved with growth arrest and apoptosis as suggested previously [53].

We identified two breast cancer cell lines, SKBR3 and MDA-MB468 that are unusually sensitive to ING1-induced cell death. Both lines have mutant p53 and negative ER status, features that are characteristic of more advanced stages of breast cancer. This suggests that ING1 may preferentially target more aggressive tumors (5). However, other breast cancer cell lines which have p53 and ER status similar to SKBR3 and MDA-MB468, exhibited a more limited degree of apoptosis upon ING1b and ING2 overexpression indicating that other factors in the two susceptible lines contribute to their sensitivity to ING-induced apoptosis. It is worth noting that the two most susceptible lines also expressed relatively higher levels of ING1 compared to other cancer lines (Figure S1 A), but how this might be related to sensitivity is currently unknown. Future experiments to compare the gene expression profiles in response to ING1 expression in susceptible versus resistant breast cancer lines will help determine apoptotic pathways impinged upon by the ING1 protein. It may also be informative to examine other components of known ING complexes such as the members of the HDAC complexes that INGs 1 and 2 participate in [9] to understand the differential sensitivity of the lines to overexpression of the INGs.

When used together, treatment of cells with 5azaC and LBH589 showed an effect greater than when each compound was used individually, but effects were less than additive suggesting antagonism, an idea borne out by high CI values on the isobologram shown in Figure 3A. In contrast, ING1b showed synergy with 5azaC and with LBH589, but significantly greater synergy was seen with 5azaC as depicted by lower CI values (compare Figures 3B&C). While the most likely explanation for this difference is that targeting two different epigenetic pathways is more effective than targeting one pathway with two agents, this does not explain why ING1b plus 5azaC is so much more effective that 5azaC and LBH589 in inducing apoptosis. One explanation may be that the mechanism of ING1 synergy with 5azaC may go beyond effects by ING1 upon histone acetylation, consistent with our preliminary results in a separate study showing that INGs also have extranuclear effects upon apoptosis.

To further address the underlying mechanism of this enhanced apoptosis and cell death, the expression and processing of caspase-3 and PARP were examined. Both caspase-3 and PARP are cleaved in the late “execution” apoptotic phase and the combination of ING1b and 5azaC synergistically induced cleavage of caspase 3. ING1b also synergistically enhanced the ability of 5azaC to induce DNA damage which, when added together with activation of effectors of apoptosis, may explain the enhanced apoptosis and cell death induction by the combination of ING1b and 5azaC.

In our *in vivo* experiments, we demonstrate that a combination of ING1b plus 5azaC could suppress the growth of subcutaneously xenografted tumors in SCID mice without any significant toxicity as determined by maintenance of body weight (Figure S6). Moreover, the tumors did not develop any resistance when the treatment was stopped and responded well on the reintroduction of the combination, particularly when Ad-ING1 virus was used at a concentration optimized *in vivo*. This suggests the potential for using ING1 as a novel therapeutic agent since it enhances the efficacy of chemotherapeutic drugs when used in combination in breast cancer patients. It is also worth mentioning that MDA-MB468 is a breast cancer cell line that is triple negative (ER−/PR−/HER2-), which would typically be derived from an advanced, aggressive and metastasized clinical stage tumor. Such tumors are insensitive to treatments such as hormone therapy or Herceptin targeted therapy and if they become resistant to chemotherapy, there are no known treatments that are able to ameliorate this disease. Given the effective nature of targeting the two epigenetic pathways shown in this study, we propose that this strategy may provide an effective therapeutic approach for cancer patients who have exhausted other modes of treatment.

## Supporting Information

Figure S1 **Relative levels of ING1b and ING2.** **A**) Western blot of cells lines before and after infection with AdING1b/AdING2. The same number of cells (1×10^5^) from all four cell lines were plated and infected with 10 MOI of AdING1b or AdING2. Cells were harvested 24 hours post infection and levels of ING1b and ING2 were analyzed by western blotting. All four cell lines show equal amount of ING1b and ING2 induction after infection with adenoviral constructs. **B**) Cell lines after infection with AdING1b/AdING2. Same number of cells (1×10^5^) from all four cell lines were plated and infected with 10 MOI of AdING1b or AdING2. Images were taken 24 hours post adenoviral infection. All four cell lines show equal amount of infection rate as determined by GFP expression.
(TIF)

Figure S2 **Growth curves of breast cancer cell lines examined.** The ten established breast cancer cell lines indicated and the primary breast epithelial strain Bst578 were grown in the media indicated in Materials & Methods. Following the plating of 5×10^5^ cells and their recovery for 12 hours (time 0), plates of cells were trypsinized and counted by Coulter counter at 0, 24 and 48 hour time points. The fastest growing cells, BT20, doubled every 24 hours while normal Bst578 cells doubled in ∼72 hours.
(TIF)

Figure S3 **Cell death in T47D cells in response to ING1b and epigenetic chemotherapeutics.** T47D cells were grown and treated with various concentrations of **A**) LBH589 and 5azaC alone or in combination, or **B,C**) in combination with adenoviral constructs expressing GFP plus ING1b at various MOIs. MOIs used were significantly greater than for MDA-MB468. The levels of cell death induced by these treatments were estimated by MTT assay. The combination of 5azaC with ING1b shown in panel C was more effective in inducing cell death in T47D cells compared to other agents tested singly or in combination.
(TIF)

Figure S4 **Combination Indices of ING1b with epigenetic chemotherapeutics.** T47D cells were treated with combinations of **A**) LBH589 plus 5azaC, **B**) adenoviral vector expressing GFP plus ING1b and LBH589 or **C**) adenoviral vector expressing GFP plus ING1b plus 5azaC at various concentrations and Combination Indexes were determined using CalcuSyn software. The Isobologram analysis showed that 5azaC plus Ad-ING1b showed the highest degree of synergy in inducing cell death of the combinations tested.
(PDF)

Figure S5 **Annexin V/7AAD staining determined by flowcytometry.** Raw data from flow cytometer showing annexin V and 7AAD dual staining of cells.
(TIF)

Figure S6 **5azaC dose response study.** Based upon literature values we tested three doses of 5azaC for their ability to **A**) affect tumor growth and **B**) impact animal growth. An intermediate dose of 5 mg/kg body weight was found to affect tumor growth without affecting body weight and since it had no other observed deleterious effects on test subjects it was chosen as the test concentration to be used in subsequent experiments.
(TIF)

Figure S7 **Apoptosis and cell death in response to ING1b and 5azaC.** **A**) Scatter plots of intact (red) and dead (black) cells as estimated by flow cytometry. Cells staining for annexin V were deemed apoptotic. **B**) Total dead and apoptotic cells. Total dead cells were calculated for the control assay and this value was subtracted from the sums of apoptotic and dead cells for all other treatments.
(TIF)

Figure S8 **Quantitation of caspase 3 and PARP cleavage.** **A**) Relative caspase cleavage in response to treatment. Bars represent the mean and SD of three scans and all other values were compared to the control which was set as 1. **B**) Relative PARP cleavage compared to control untreated cells. Values are the mean and SD of three scans.
(TIF)
