# Supplementary figures and images for: ING1 and 5-Azacytidine Act Synergistically to Block Breast Cancer Cell Growth
Source: PLoS One. 2012 Aug 20;7(8):e43671. doi: 10.1371/journal.pone.0043671 (PMC3423394; doi:10.1371/journal.pone.0043671)

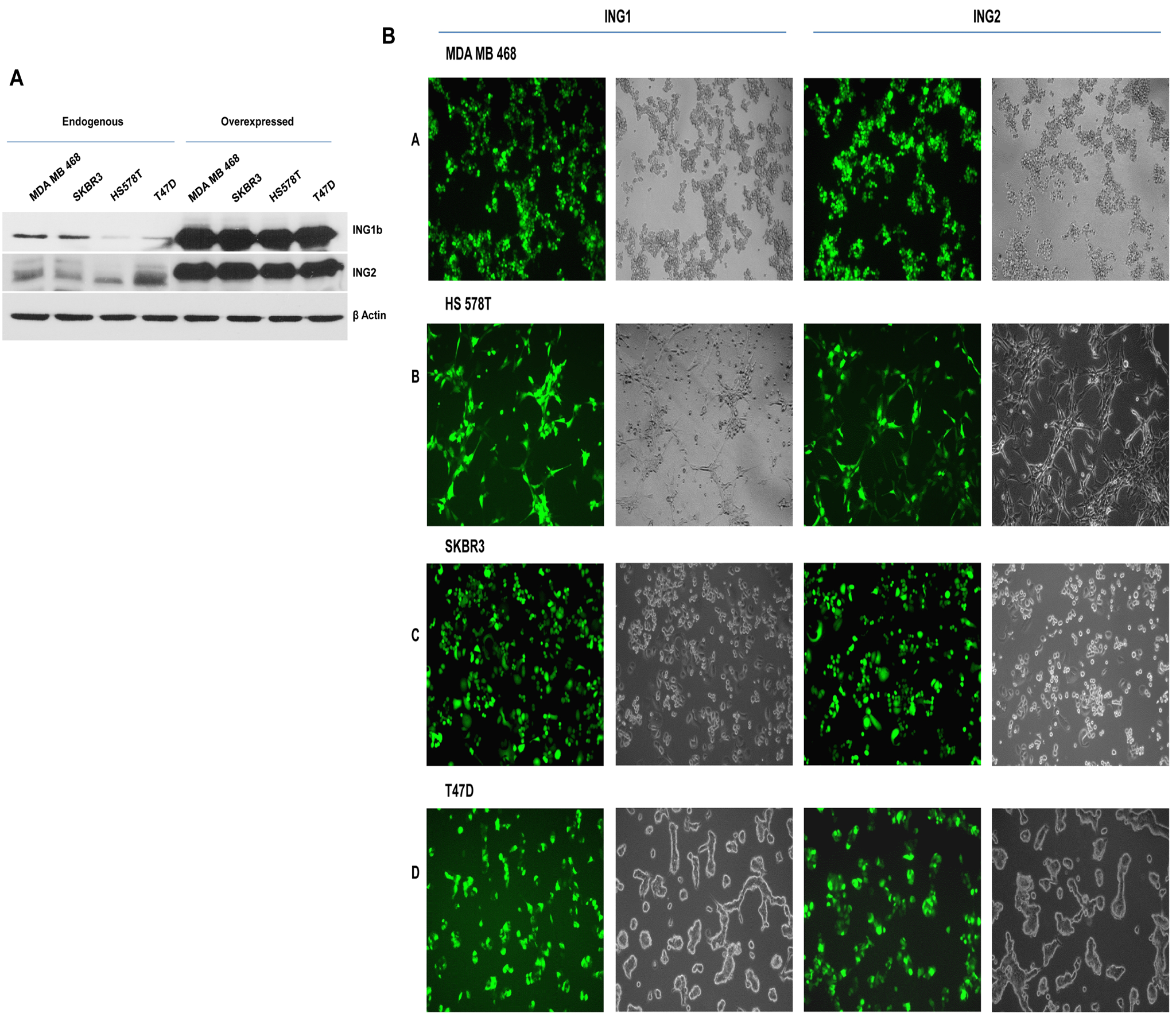

Supplement: Figure S1 — Relative levels of ING1b and ING2. A) Western blot of cells lines before and after infection with AdING1b/AdING2. The same number of cells (1×10^5^) from all four cell lines were plated and infected with 10 MOI of AdING1b or AdING2. Cells were harvested 24 hours post infection and levels of ING1b and ING2 were analyzed by western blotting. All four cell lines show equal amount of ING1b and ING2 induction after infection with adenoviral constructs. B) Cell lines after infection with AdING1b/AdING2. Same number of cells (1×10^5^) from all four cell lines were plated and infected with 10 MOI of AdING1b or AdING2. Images were taken 24 hours post adenoviral infection. All four cell lines show equal amount of infection rate as determined by GFP expression. (TIF) [file pone.0043671.s001.tif]

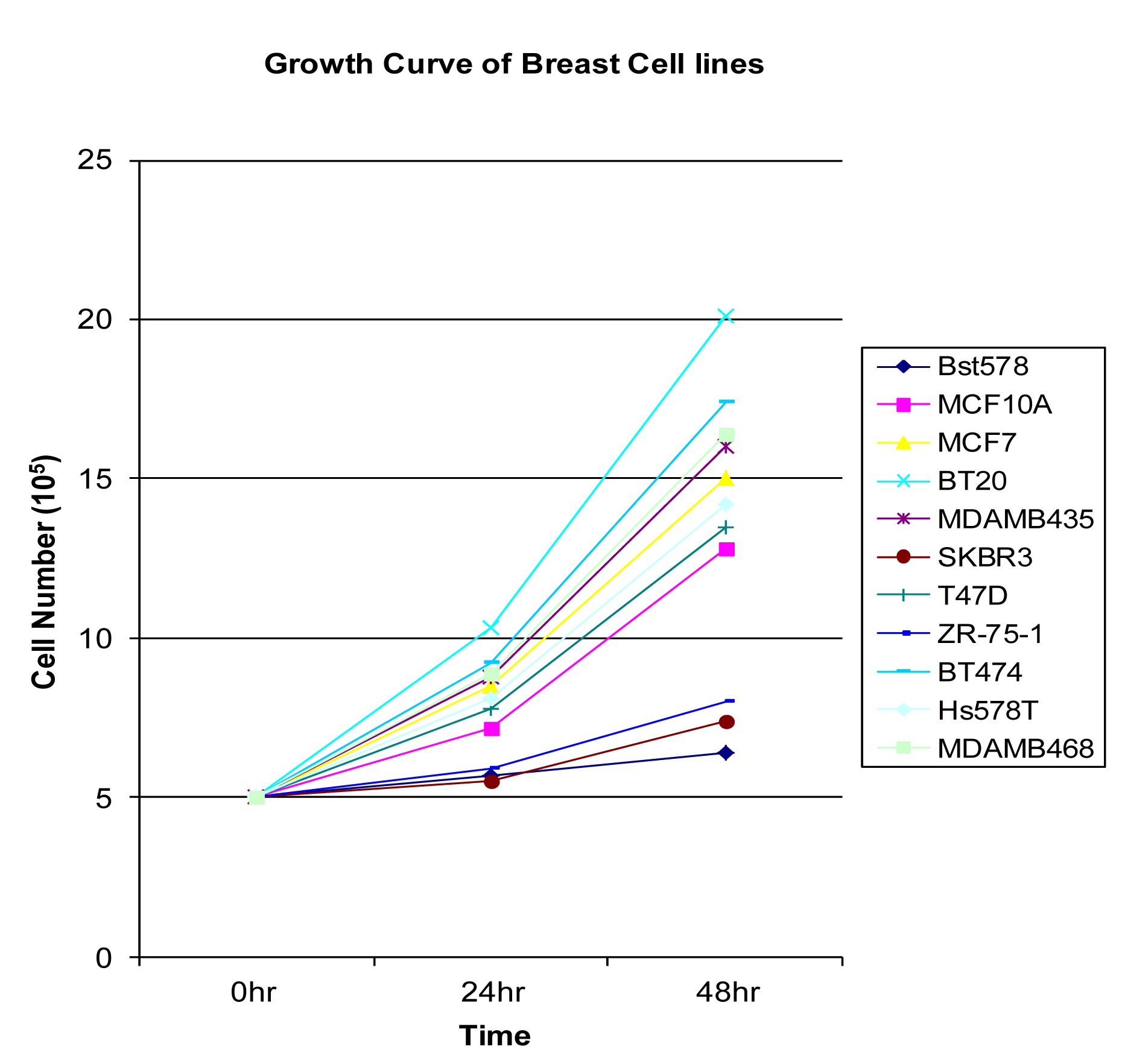

Supplement: Figure S2 — Growth curves of breast cancer cell lines examined. The ten established breast cancer cell lines indicated and the primary breast epithelial strain Bst578 were grown in the media indicated in Materials & Methods. Following the plating of 5×10^5^ cells and their recovery for 12 hours (time 0), plates of cells were trypsinized and counted by Coulter counter at 0, 24 and 48 hour time points. The fastest growing cells, BT20, doubled every 24 hours while normal Bst578 cells doubled in ∼72 hours. (TIF) [file pone.0043671.s002.tif]

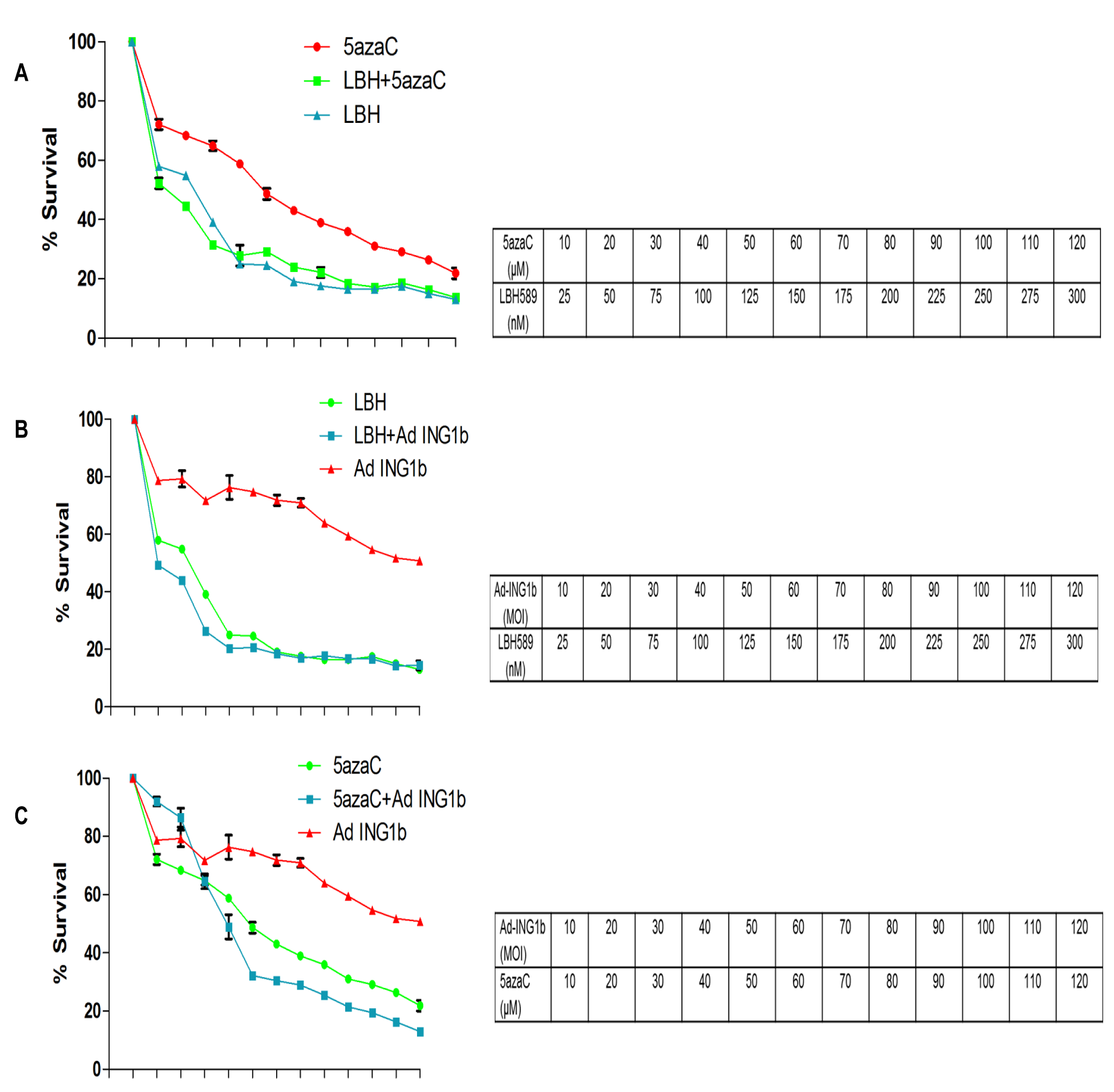

Supplement: Figure S3 — Cell death in T47D cells in response to ING1b and epigenetic chemotherapeutics. T47D cells were grown and treated with various concentrations of A) LBH589 and 5azaC alone or in combination, or B,C) in combination with adenoviral constructs expressing GFP plus ING1b at various MOIs. MOIs used were significantly greater than for MDA-MB468. The levels of cell death induced by these treatments were estimated by MTT assay. The combination of 5azaC with ING1b shown in panel C was more effective in inducing cell death in T47D cells compared to other agents tested singly or in combination. (TIF) [file pone.0043671.s003.tif]

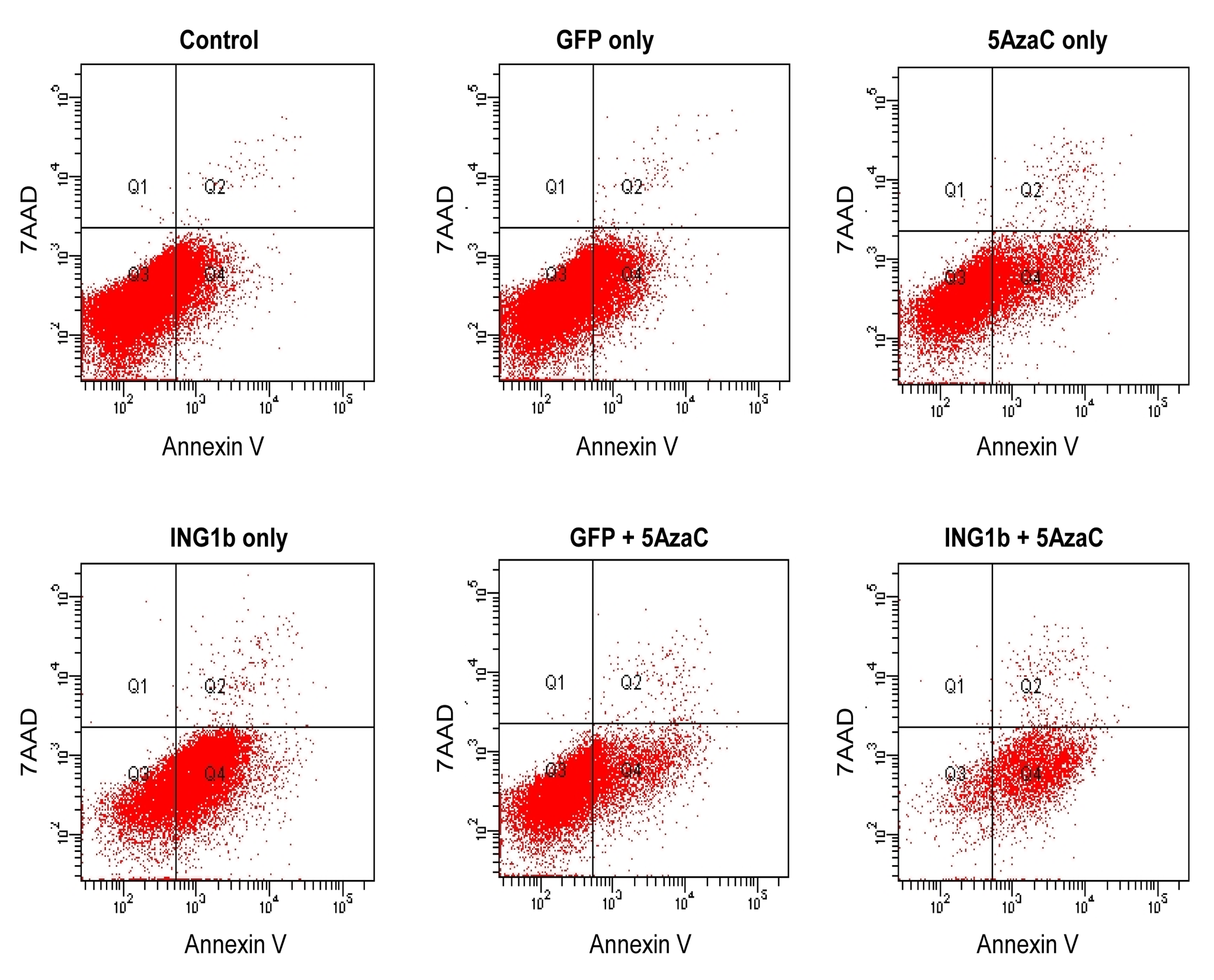

Supplement: Figure S5 — Annexin V/7AAD staining determined by flowcytometry. Raw data from flow cytometer showing annexin V and 7AAD dual staining of cells. (TIF) [file pone.0043671.s005.tif]

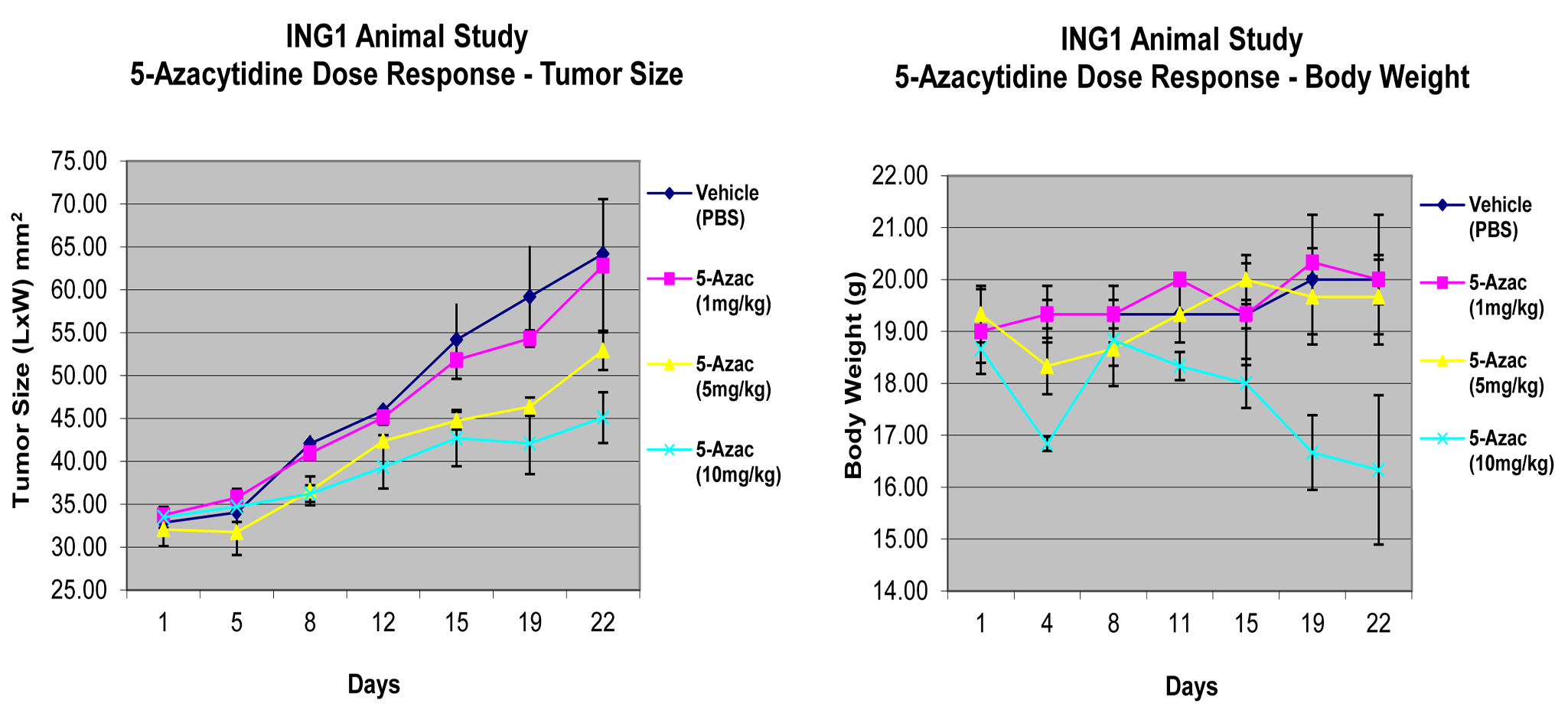

Supplement: Figure S6 — 5azaC dose response study. Based upon literature values we tested three doses of 5azaC for their ability to A) affect tumor growth and B) impact animal growth. An intermediate dose of 5 mg/kg body weight was found to affect tumor growth without affecting body weight and since it had no other observed deleterious effects on test subjects it was chosen as the test concentration to be used in subsequent experiments. (TIF) [file pone.0043671.s006.tif]

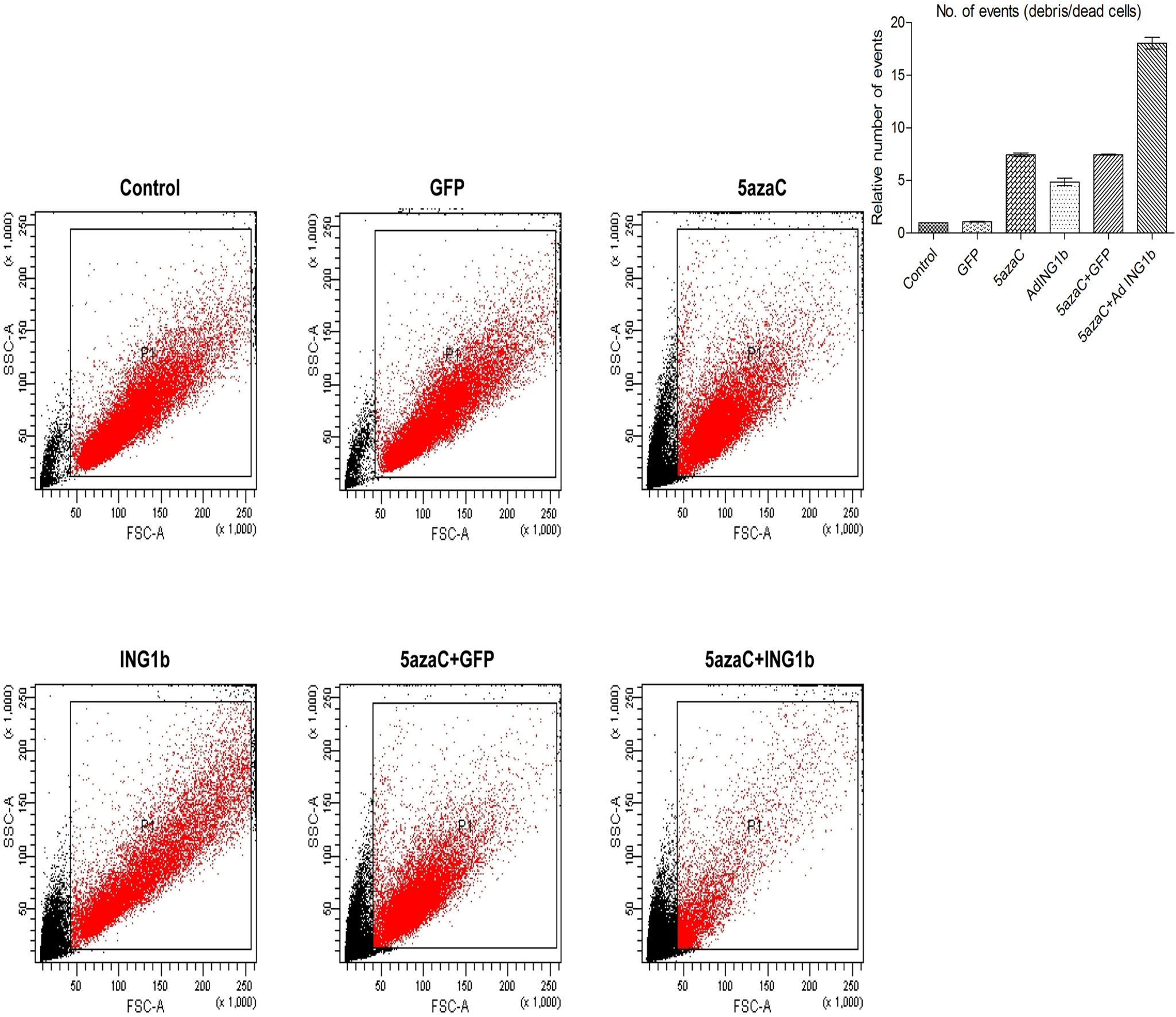

Supplement: Figure S7 — Apoptosis and cell death in response to ING1b and 5azaC. A) Scatter plots of intact (red) and dead (black) cells as estimated by flow cytometry. Cells staining for annexin V were deemed apoptotic. B) Total dead and apoptotic cells. Total dead cells were calculated for the control assay and this value was subtracted from the sums of apoptotic and dead cells for all other treatments. (TIF) [file pone.0043671.s007.tif]

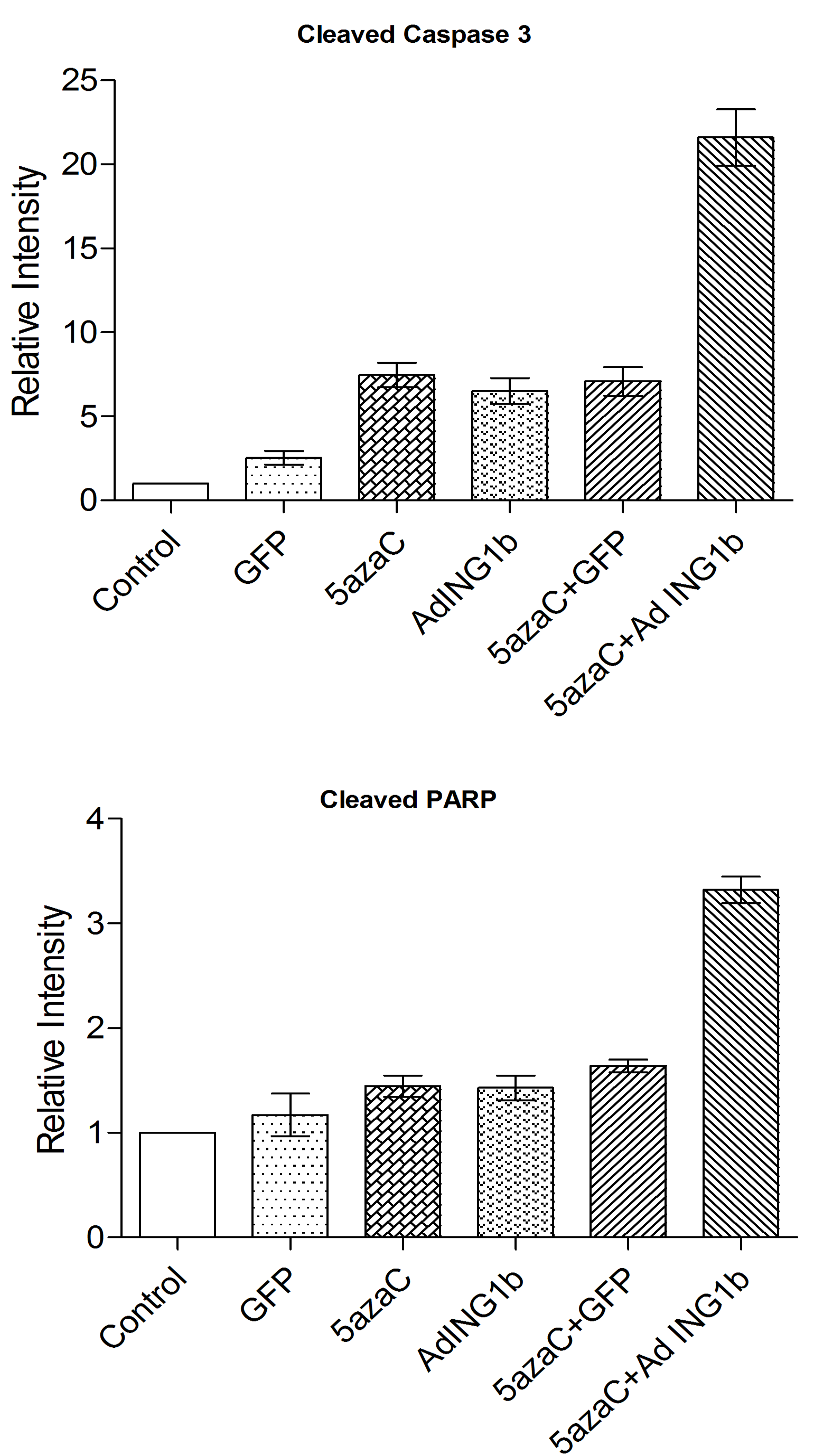

Supplement: Figure S8 — Quantitation of caspase 3 and PARP cleavage. A) Relative caspase cleavage in response to treatment. Bars represent the mean and SD of three scans and all other values were compared to the control which was set as 1. B) Relative PARP cleavage compared to control untreated cells. Values are the mean and SD of three scans. (TIF) [file pone.0043671.s008.tif]
