# Supplementary material for: ING1 and 5-Azacytidine Act Synergistically to Block Breast Cancer Cell Growth
Source: PLoS One. 2012 Aug 20;7(8):e43671. doi: 10.1371/journal.pone.0043671 (PMC3423394; doi:10.1371/journal.pone.0043671)
Supplement: Figure S4 — Combination Indices of ING1b with epigenetic chemotherapeutics. T47D cells were treated with combinations of A) LBH589 plus 5azaC, B) adenoviral vector expressing GFP plus ING1b and LBH589 or C) adenoviral vector expressing GFP plus ING1b plus 5azaC at various concentrations and Combination Indexes were determined using CalcuSyn software. The Isobologram analysis showed that 5azaC plus Ad-ING1b showed the highest degree of synergy in inducing cell death of the combinations tested. (PDF) [file pone.0043671.s004.pdf]

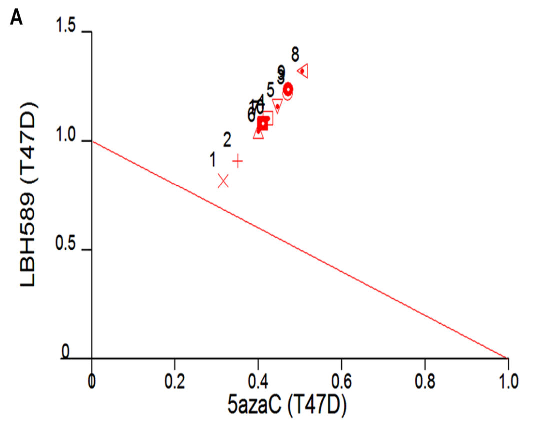


| Combination No. | LBH589 (nM) | 5azaC (μM) | CI |
|---|---|---|---|
| 1 | 75 | 30 | 1.133 |
| 2 | 100 | 40 | 1.259 |
| 3 | 125 | 50 | 1.688 |
| 4 | 150 | 60 | 1.527 |
| 5 | 175 | 70 | 1.607 |
| 6 | 200 | 80 | 1.446 |
| 7 | 225 | 90 | 1.484 |
| 8 | 250 | 100 | 1.829 |
| 9 | 275 | 110 | 1.710 |
| 10 | 300 | 120 | 1.494 |

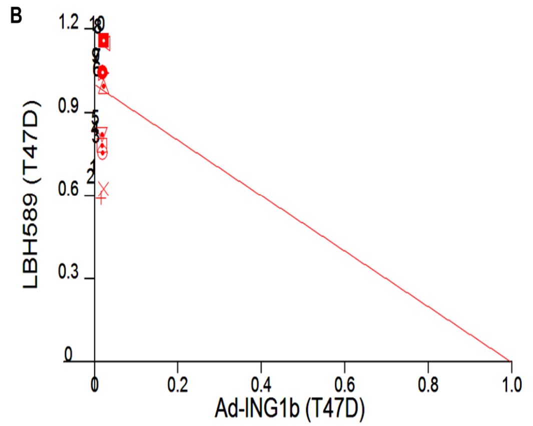


| Combination No. | LBH589 (nM) | Ad-ING1b (MOI) | CI |
|---|---|---|---|
| 1 | 75 | 30 | .648 |
| 2 | 100 | 40 | .609 |
| 3 | 125 | 50 | .775 |
| 4 | 150 | 60 | .801 |
| 5 | 175 | 70 | .839 |
| 6 | 200 | 80 | 1.017 |
| 7 | 225 | 90 | 1.065 |
| 8 | 250 | 100 | 1.174 |
| 9 | 275 | 110 | 1.064 |
| 10 | 300 | 120 | 1.180 |

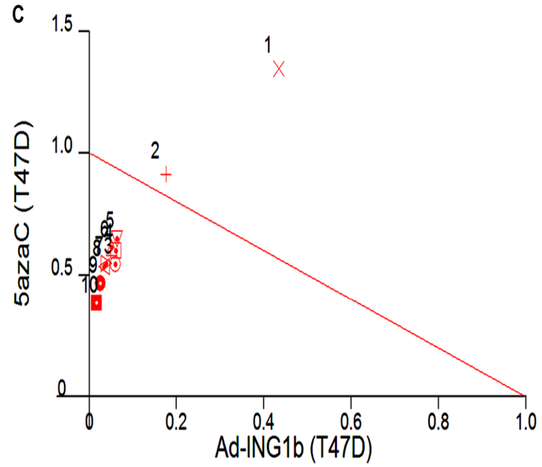


| Combination No. | 5azaC (μM) | Ad-ING1b (MOI) | CI |
|---|---|---|---|
| 1 | 30 | 30 | 1.783 |
| 2 | 40 | 40 | 1.090 |
| 3 | 50 | 50 | .604 |
| 4 | 60 | 60 | .661 |
| 5 | 70 | 70 | .714 |
| 6 | 80 | 80 | .666 |
| 7 | 90 | 90 | .587 |
| 8 | 100 | 100 | .568 |
| 9 | 110 | 110 | .494 |
| 10 | 120 | 120 | .403 |

This document was created using SOLID CONVERTER PDF
To remove this message, purchase the product at www.SolidDocuments.com
